# Spatial Co-Clustering of Cardiovascular Diseases and Select Risk Factors among Adults in South Africa

**DOI:** 10.3390/ijerph17103583

**Published:** 2020-05-20

**Authors:** Timotheus B. Darikwa, Samuel O. Manda

**Affiliations:** 1 Department of Statistics and Operations Research, University of Limpopo, Sovenga 0727, South Africa; 2 Biostatistics Research Unit, South African Medical Research Council, Pretoria 0001, South Africa; Samuel.Manda@mrc.ac.za; 3 Department of Statistics, University of Pretoria, Hatfield 0083, South Africa; 4 School of Mathematics, Statistics and Computer Science, University of KwaZulu-Natal, Pietermaritzburg 3201, South Africa

**Keywords:** cardiovascular diseases, stroke, heart attack, smoking, hypertension, high cholesterol, bivariate spatial autocorrelation, prevalence, cluster, South Africa

## Abstract

*Background:* Cardiovascular diseases (CVDs) are part of the leading causes of mortality and morbidity in developing countries, including South Africa, where they are a major public health issue. Understanding the joint spatial clustering of CVDs and associated risk factors to determine areas in need of enhanced integrated interventions would help develop targeted, cost-effective and productive mediations. We estimated joint spatial associations and clustering patterns of 2 CVDs (stroke and heart attack) and 3 risk factors (hypertension, high blood cholesterol (HBC) and smoking) among adults in South Africa. *Methods:* We used cross-sectional secondary adult (15–64-year olds) health data from the South African Demographic Health Survey 2016. Age and gender standardized disease incidence ratios were analyzed using joint spatial global and local bivariate Moran’s Index statistics. *Results:* We found significantly positive univariate spatial clustering for stroke (Moran; s Index = 0.128), smoking (0.606) hypertension (0.236) and high blood cholesterol (0.385). Smoking and high blood cholesterol (0.366), smoking and stroke (0.218) and stroke and high blood cholesterol (0.184) were the only bivariate outcomes with significant bivariate clustering. There was a joint stroke-smoking local “hot spots” cluster among four districts in the urban western part of the country (City of Cape Town; Cape Winelands; Overberg and Eden) and a joint “cold spots” cluster in the rural north-western part of the country. Similar joint “hot spots” clustering was found for stroke and high blood cholesterol, which also had “cold spots” cluster in the rural east-central part of the country. Smoking and high blood cholesterol had a “hot spots” cluster among five districts in the urban western part of the country (City of Cape Town; Cape Winelands; Overberg; Eden, and West Coast) and “cold spots” around the rural districts in east-southern parts of the country. *Conclusions:* Our study showed that districts tended to co-cluster based on the rates of CVDs and risk factors, where higher rates were found in urban places than in rural areas. These findings are suggestive of a more contagious and spatial diffusion process among interdependent districts in urban districts. Urbanization or rurality needs to be considered when intervention initiatives are implemented with more general approaches in rural areas. The finding of “hot spot” co-clusters in urban areas means that integrated intervention programmes aimed at reducing the risk of CVDs and associated risk factors would be cost-effective and more productive.

## 1. Introduction

Cardiovascular diseases (CVDs) are a predominant contributor to the world’s disease burden. In total, non-communicable diseases (NCDs) contribute the highest proportion (72%) of deaths in the world [1]. In 2016, there were 39.5 million deaths attributed to NCDs, representing a 16% growth on 2006 deaths, of which 80% were due to CVDs [1]. Ischaemic heart disease (IHD), stroke and heart failure (HF) contribute about 80% of all CVD deaths [1,2,3]. Several studies in sub-Saharan Africa (SSA) have found a high prevalence of CVD risk factors such as high levels of tobacco smoking, harmful alcohol consumption, obesity, diabetes, high blood cholesterol and high blood pressure among the adult population [4,5,6,7,8]. Sub-Saharan Africa (SSA) is projected to bear a disproportionately high burden of CVDs and associated risk factors is [9,10,11].

Several risk factors of CVD have been identified over the years. Some of these are modifiable and others are not modifiable. Modifiable risk factors, including high blood pressure (hypertension); overweight or obesity; high blood cholesterol levels; low physical activity; harmful tobacco use; harmful alcohol use; low salt intake; low fruit intake; and low vegetable intake, are essential in the prevention and management of CVDs. On the other hand, the uncontrollable risk factors such as gender, age, ethnicity, educational level, employment status, location of residence, family history and poverty status are less manageable. Evidence has shown that men are at a greater risk of coronary heart diseases (CHD) than females and the risk increases with age [12]. High level of smoking, harmful alcohol consumption, obesity, physical inactivity, high blood pressure, diabetes, and high blood cholesterol have been associated with an increased incidence of cardiovascular diseases [13,14,15,16,17,18,19].

South Africa has one of the highest burdens of cardiovascular diseases in the region [20,21]. This has been fueled by rapid urbanization and changes in lifestyle, which are more sedentary, and high salt and fat and sugar diet dependency [22,23]. The country currently spends a quarter of its total public health budget (or 3% of its gross domestic product) containing CVDs [24]. However, resources allocated to containing CVDs are constrained, with a high emphasis being placed on alleviating the problem of human immunodeficiency viruses/ acquired immunodeficiency syndrome (HIV/AIDs) and other infectious diseases [25]. South Africa is a signatory to the World Health Organization’s (WHO) Sustainable Development Goals (SDGs), including number 3 [26], which tasks governments to proactively monitor, prevent and control NCDs. South African National Department of Health (NDoH) strategies aimed at reducing NCD morbidity, mortality and associated risk factors have identified CVDs as a priority [27].

This study was set up to empirically analyse the extent of spatial dependence at both the national and local level regarding cardiovascular diseases and related risk factors in South Africa. Results of the study will help local and national policymakers to develop more relevant programmes and interventions as part of the National NCD strategies and plans. Our study will help to identify districts of high CVD risk to be prioritized in the provision of primary health care in line with recommendations by Schutte [25]. Few studies have looked at the geographic distribution of CVD and risk factors in the country. For example, Kandala et al. [28,29] used spatial modelling to estimate the geographic variation of CVD and risk factors; however, the data used were from a cross-sectional survey done in 1998. Wandai et al. [30] described the district levels of hypertension in South Africa. All three studies found variations in the risk of CVD and risk factors. In our study, we use different spatial analysis approaches to measure clustering at both national and local levels for multiple CVDs and risk factors simultaneously. The numbers of CVD diseases and risk factors have also been expanded to include stroke, heart attack, hypertension, high blood cholesterol (HBC) and smoking among adults in South Africa. Our approach is like that described in Darikwa et al. [31] for the analysis of CVD-related deaths in South Africa. Furthermore, rather than using observed rates, our approach is based on age–gender standardized rates to remove the cofounding effects of age and gender in comparing the spatial clustering patterns. Evidence suggests that CVDs and risk factors co-vary in space [29]. Thus, estimating joint “hot spots” and “cold spots” clusters of districts for two or more CVDs will provide more evidence for an integrated intervention approach that targets all CVDs.

## 2. Materials and Methods

### 2.1. Data

**Figure 1:**
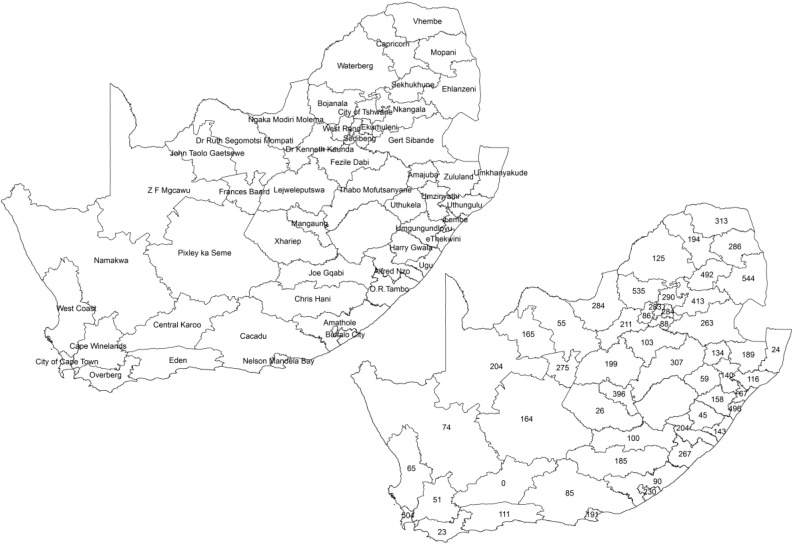
The South African map showing the district names and sample sizes: South African Demographic and Health Survey, 2016.

This study used secondary data collected as part of the South Africa Demographic and Health Survey in 2016 (SADHS 2016). The SADHS 2016’s adult health module recorded information that included, among others, the self-reported prevalence of two CVDs, stroke and heart attack, and the three risk factors of interest: raised blood pressure, raised cholesterol and smoking for both male and female adults aged 15 years and older. A total of 12,717 adults were targeted for this adult health module, but 10,336 responded. We used health districts for the spatial analysis in this study. Figure 1 shows the map of the 52 districts of South Africa and the number of the sampled adults, which ranged from 23 to 544 per district, with an average of 203 subjects. Due to the sample design, Central Karoo, which has a very sparse population was not sampled from and was not included in the analyses. For the purposes of our study, the data were stratified by gender (male and female) and age (15–39 years (young adults) and 40–64 years (adults)). A cut-off point of 40 years was used as it has been observed that the burden of CVDs increased significantly after the age of 40 years [32,33].

### 2.2. Variable Definitions

The CVD variables considered in this study were stroke and heart attacks and are defined below.

- Stroke: a dichotomous variable in which a person who self-reported to have been diagnosed with stroke is a success and is assigned a value 1 and zero otherwise.
- Heart attack: a dichotomous variable in which a person who self-reported to have been diagnosed with a heart attack was assigned a value 1 and zero otherwise.
- Three risk factors of CVDs considered in this study are smoking, hypertension and high blood cholesterol. These are defined below.
- Smoking: a binary variable in which a respondent who stated that he/she smokes daily or occasionally is assigned a value 1 and zero otherwise.
- High blood cholesterol: a dichotomous variable in which a person who self-reported to have been diagnosed with high cholesterol is a success and is assigned a value 1 and zero otherwise.
- Hypertension: this was defined as a systolic BP measurement of at least 140 mmHg or diastolic BP measurement of at least 90 mmHg or self-report of hypertension diagnosis as hypertensive or on hypertension medication.

### 2.3. Statistical Methods

Preliminary analyses involved calculating pairwise correlations between the prevalence data of the two CVDs and their risk factors. Both univariate and bivariate Moran’s index of spatial autocorrelation were used to assess univariate and bivariate spatial dependence among the five CVD diseases and risk factors. The queen’s spatial contiguity weight matrix was used.

Regarding the standard Moran’s *I*, we suppose $w_{ij}$ are spatial weight taking a value of 1 or 0 depending on whether districts i and j are neighbours or not. Denoting the prevalence of a disease by $y_{i},$ the global Moran’s *I* is defined as:

(1) $$I=\frac{n}{\sum_{i=1}^{n}\sum_{j=1}^{n}w_{ij}}\frac{\sum_{i=1}^{n}\sum_{j=1}^{n}w_{ij}(y_{i}-\bar{y})(y_{j}-\bar{y})}{\sum_{i=1}^{n}{\left(y_{i}-\bar{y}\right)}^{2}}\;\;\;\;,\;\;\;i\neq j$$

where n is the number of districts in South Africa. The corresponding local univariate Moran’s *I* is then defined by:

(2) $$I_{i}=\frac{n(y_{i}-\bar{y})\times \sum_{j=1}^{n}w_{ij}(y_{j}-\bar{y})}{\sum_{j=1}^{n}{\left(y_{j}-\bar{y}\right)}^{2}}\;\;\;\;,\;\;\;i\neq j\;\;\;\;\;\;\;$$

where $\frac{1}{n}\sum_{i=1}^{n}I_{i}=I.$

Based on the original work of Mantel [33], the univariate Moran’s *I* has recently been expanded to cases where there is more than one spatially aligned measurement. A brief formulation of bivariate is given below. For two spatially dependent disease outcomes, say $y^{\left(1\right)}$ and $y^{\left(2\right)}$, the global bivariate Moran’s index is given by:

(3) $$I=\frac{n}{\sum_{i=1}^{n}\sum_{j=1}^{n}w_{ij}}\frac{\sum_{i=1}^{n}\sum_{j=1}^{n}w_{ij}(y_{i}^{\left(1\right)}-{\bar{y}}^{\left(1\right)})(y_{j}^{\left(2\right)}-{\bar{y}}^{\left(2\right)})}{\sum_{i=1}^{n}{\left(y_{i}^{\left(1\right)}-{\bar{y}}^{\left(1\right)}\right)}^{2}}\;\;\;\;,\;\;\;i\neq j$$

and the corresponding local bivariate Moran’s *I* is defined by [34]:

(4) $$I_{i}=\frac{n(y_{i}^{\left(1\right)}-{\bar{y}}^{\left(1\right)})\times \sum_{j=1}^{n}w_{ij}(y_{j}^{\left(2\right)}-{\bar{y}}^{\left(2\right)})}{\sum_{j=1}^{n}{\left(y_{j}^{\left(2\right)}-{\bar{y}}^{\left(2\right)}\right)}^{2}}\;\;\;\;,\;\;\;i\neq j\;\;\;\;\;\;\;$$

where $\frac{1}{n}\sum_{i=1}^{n}I_{i}=I$.

The estimation of both the global bivariate spatial autocorrelations and joint local clusters of districts were implemented in spatial analysis software GeoDa [35].

## 3. Results

### 3.1. Descriptive Statistics of the Variables and Their Correlations

**Figure A1:**
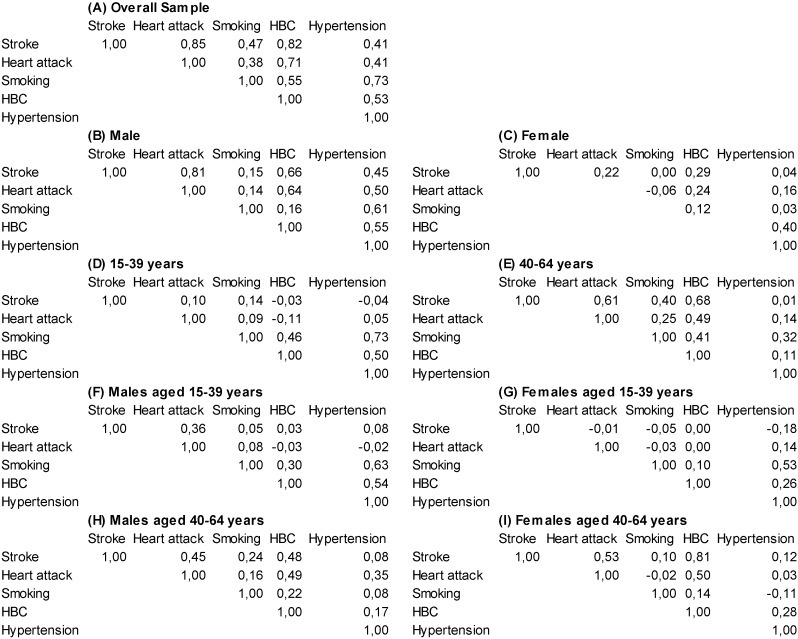
Correlations between CVDs and their risk factors.

A total of 9154 participants aged between 15 and 64 years were sampled, of which 5337 (58%) were females and 5848 (64%) were aged between 15 and 39 years. The overall mean prevalence of stroke, heart attack, high blood cholesterol, hypertension and smoking was 1.20%; 95% CI (0.77%, 1.63%), 2.40% (1.85%, 2.95%), 2.45% (1.57%, 2.93%), 34.28% (31.37%, 37.19%) and 21.90% (19.14%, 24.66%), respectively. We also summarized the data at the level of the district, and these are shown in Table 1 by the overall adult sample, gender and age groups. District level prevalence ranges from 0% to 100% across the CVDs and related risk factors. On average, the prevalence of heart attack (2.4%) is twice the prevalence of strokes (1.2%). The prevalence of heart attack was higher in females (2.8%) than among males (1.8%). District-level prevalence of smoking has an average of 22%. Smoking was higher among males (39%) than in females (10%). The correlation between district-level raw prevalence between stroke and heart attack(0.85); stroke and HBC (0.82); heart attack and HBC (0.71); smoking and HBC (0.55); smoking and hypertension (0.73); and HBC and hypertension (0.53) (See Figure A1 in the Appendix A).

**Figure 2:**
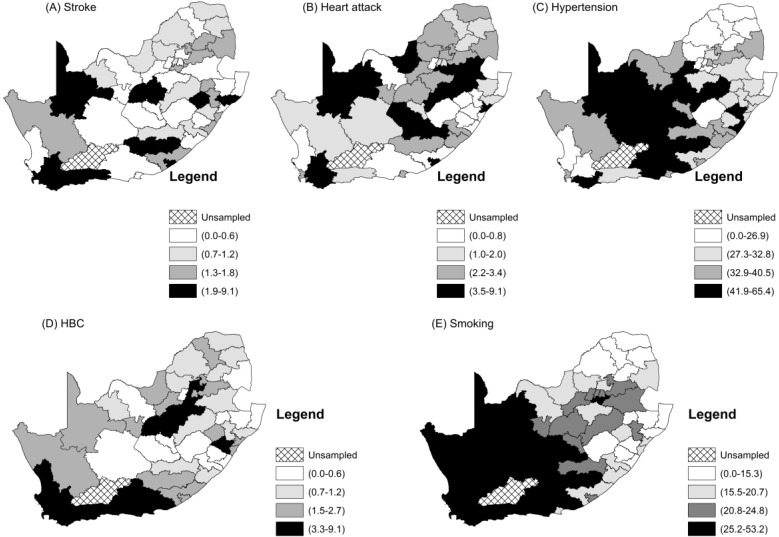
Maps of the district prevalence rates of the CVDs and their related risk factors.

**Figure 3:**
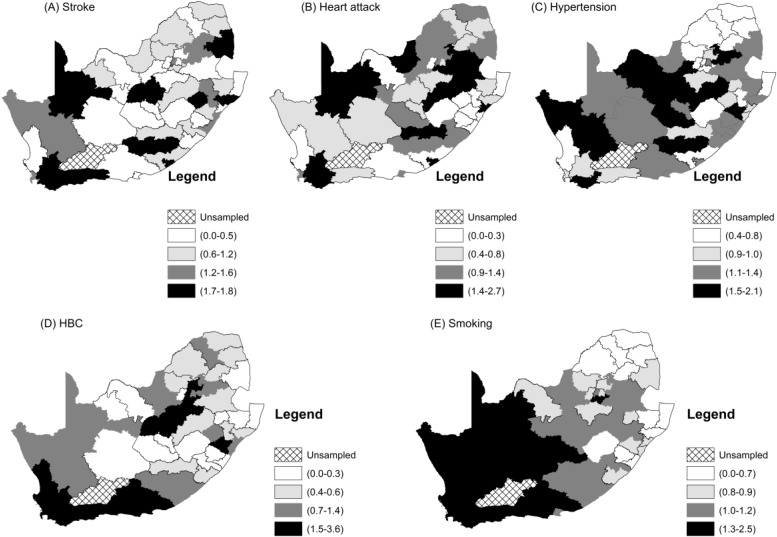
Maps of the district standardized incidence rates of the CVDs and their related risk factors.

Figure 2 and Figure 3 show the raw observed prevalence rate and standardized incidence ratio by district. Lower rates of all the two CVDs and three risk factors were seen in the more rural upper north-east of the country, while higher rates of smoking and high blood cholesterol were observed in the more south-western parts. All five CVD measures were higher in the more urban areas of the western part of the country, even though stroke and heart attack showed an even fluctuation. Higher rates of hypertension were more concentrated in the middle-western part of the country.

### 3.2. Joint Spatial Clustering Analysis

In our analyses, we could have used the raw prevalence of the two cardiovascular diseases and the three associated risk factors. However, the estimated level of spatial clustering would be misleading because of confounders such as age and gender that have an important effect on CVDs and their risk factors. We considered calculating age–gender-adjusted prevalence; however, the district age-gender specific prevalence would be less reliable and unstable because of smaller district samples and observed cases observations, which resulted in a huge amount of random error (See Table A1 (Appendix B)). On the other hand, age-gender-specific prevalence calculated from the overall adult sample should be much more stable, because of the larger sample size. In this study, we used the age–gender specific prevalence obtained from whole SADHS adult (15–64 years) to estimate the expected number of CVD and risk factor cases based on the age–gender-distribution of each district to obtain standardized incidence ratios (SIR). The SIR is simply a ratio of the observed number of cases of a condition divided by the expected number of cases. We use SIRs here for the main bivariate spatial autocorrelation analyses.

#### 3.2.1. Estimates of Univariate and Bivariate Moran’ Measure of Spatial

The values of univariate and bivariate measures of spatial clustering are presented in Table 2, where the diagonal values are the univariate global Moran’s *I*. The off-diagonals are the global bivariate spatial autocorrelation indexes for the association between the SIRs of CVDs and identified risk factors. There is no evidence of spatial dependence between heart attack and all the three risk factors of CVDs at 5% significance level. Stroke is significantly spatially associated with smoking and HBC. In addition, there is also a high spatial dependence between smoking and HBC (*p*-value less than 0.001).

**Figure A2:**
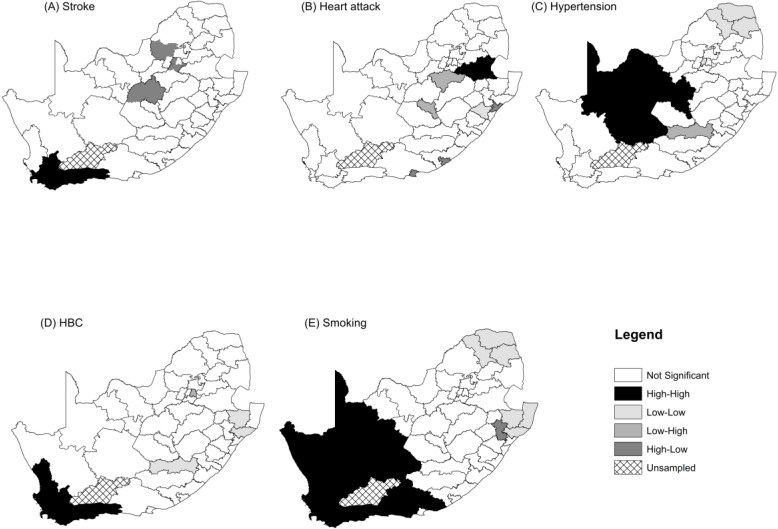
Univariate LISA cluster for the standardized incidence ratios of stroke, heart attack, hypertension, high blood cholesterol and Smoking.

**Figure A3:**
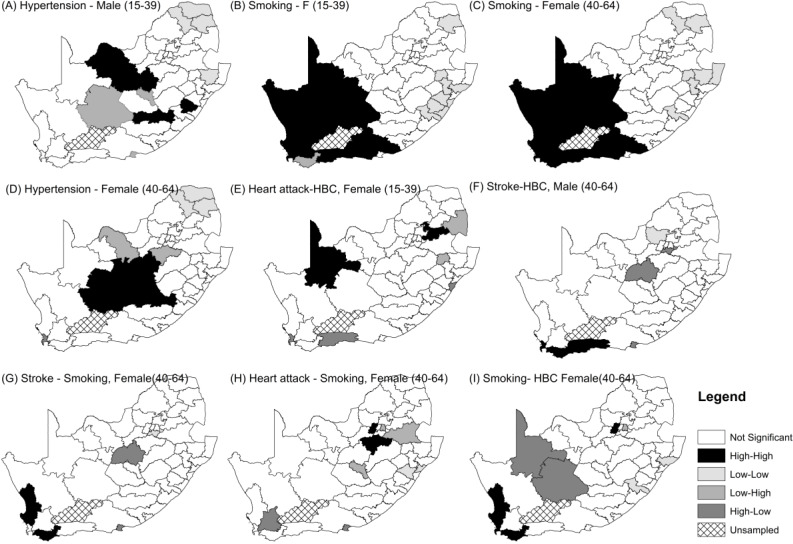
Spatial Clusters of Prevalence of five CVDs and their risk factors.

We also estimated univariate local indicators of spatial autocorrelations (LISA) for the five CVDs and risk factors. These are shown in Figure A3. Clusters of a high prevalence of smoking in districts that are surrounded by districts with a high prevalence of smoking are in Figure A3E. They form the largest “hot spots” cluster in the western part of the country. Ten districts constitute this “hot spots” cluster. These are Cacadu (Eastern Cape Province), Namakwa (Northern Cape), Pixley ka Same (Northern Cape), ZF Mgcawu (Northern Cape), Frances Baard (Northern Cape), City of Cape Town (Western Cape), West Coast (Western Cape), Overberg (Western Cape), Cape Winelands (Western Cape) and Eden District (Western Cape). There are some “cold spots” clusters of smoking that are comprised of Zululand, Uthungulu, Umkhanyakhude (all in KwaZulu-Natal Province), and Capricorn and Mopani District (in Limpopo Province). These “cold spots” are generally clustered around rural districts. Hypertension has a “hot spots” cluster that is made up of seven districts, namely Xhariep, Lejweleputswa, Mangaung (all in Free State Province), Pixley ka Seme, ZF Mgcawu, Frances Baard, and Dr Ruth Segomotsi District (North West). The cold spots are comprised of Capricorn, Vhembe, Mopani (Limpopo) and Johannesburg District in Gauteng Province. The “hot spots” clusters of stroke and HBC in Maps A and D of Figure A2 respectively, are concentrated in the Western Cape Province. They both share the “hot spots” districts of City of Cape Town, Eden, Overberg and Cape Winelands. In addition, the “hot spots” cluster of HBC includes West Coast District. The global univariate Moran index for heart attacks was not significant, but we included the LISA map shown in Map B Figure A2. It shows a significant “hot spot” of one district called Gert Sibande in Mpumalanga and a “cold spot” in Umgugundlovu in KwaZulu Natal.

**Figure 4:**
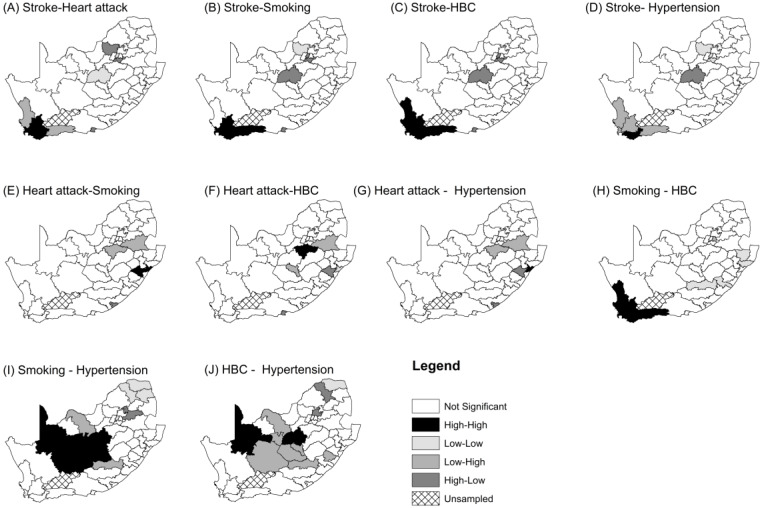
Joint spatial clusters of CVDs and their risk factors with significant association for all participants, adjusted for the national age-sex distribution of the sample.

Figure 4 shows local joint clusters for different pairwise CVD and risk factors. A joint stroke-smoking “hot spots” cluster of districts (comprising West Coast, City of Cape Town, Cape Winelands, Overberg and Eden) was found in the south-western part of the country. A similar joint “hot spots” cluster was found for stroke and HBC, and for smoking and HBC (Maps B and C in Figure 4). A joint “hot spots” cluster of smoking and HBC is also concentrated in the Western Cape Province and is comprised of West Coast, City of Cape Town, Cape Winelands, Overberg and Eden Districts. The following “cold spots” were observed for significant associations: stroke and smoking, in Bojanala (rural North West Province); stroke and HBC, in Sedibeng (rural Gauteng Province), West Rand (urban and rural Gauteng Province), and Lejweleputswa (rural Free State Province); and smoking and HBC, Alfred Nzo, Joe Gqabi (rural Eastern Cape Province), Zululand and UThungulu (rural KZN Province). There were bivariate associations that were not significant: heart attack and stroke; heart attack and HBC; heart attack and hypertension; heart attack and smoking; smoking and hypertension; HBC and hypertension.

#### 3.2.2. Clustering Using Observed Prevalence Data

As a sensitivity analysis, we also approached the analyses using observed prevalence data within age–gender grouping, namely males aged 15–39 years; females aged 15–39 years; males aged 40–64 years; and females aged 40–64 years. The results are presented in Figure A3, where significant univariate clusters and bivariate co-clusters were identified. Males had only one co-cluster: Stroke and HBC. This was for males aged 40–64 years in the City of Cape Town. There are three co-clusters for females aged 40–64 years: stroke-smoking; HA-smoking; and HBC-smoking. These are in Overberg and West Coast. Females aged 15–39 have clusters for heart attack-HBC.

## 4. Discussion

Our study aimed to measure the joint spatial clustering of two cardiovascular diseases, namely stroke and heart attack, and three cardiovascular risk factors, namely tobacco smoking, hypertension and high blood cholesterol in South Africa. This was accomplished by applying global and local bivariate Moran’s index on age–gender standardized rates using adult health data from the South African Demographic and Health Survey of 2016. There was evidence of spatial dependency between stroke and smoking, stroke and high blood cholesterol, and between smoking and high blood cholesterol. This revealed that there is a tendency of nearby districts to have high or low joint stroke-smoking, stroke-high blood cholesterol and smoking-high blood cholesterol indexes of spatial autocorrelation. The study established local high-high joint stroke-smoking or stroke-high blood cholesterol or smoking-high blood cholesterol in the urban districts of the western part of the country (City of Cape Town; Cape Winelands; Overberg and Eden). However, the same bivariate outcomes exhibited low-low clusters in rural north-western (for stroke and smoking), central and north-west districts (for stroke and HBC) and south-eastern parts of the country (for smoking and HBC).

Thus, this study suggests that the spatial clustering of CVDs and risk factors differs according to urbanisation or rurality locations, with urban districts having high-high district clusters and rural areas having low-low district clusters of CVDs and the risk factors. Differentials in urban and rural clustering of CVDs or their risk factors based on the values of the rates have been reported elsewhere [36,37,38,39]. In the more developed countries, for example, Sweden [39] and Canada [38], the “high-high” clustering areas of CVD or their risk factors were found in rural areas, while “low-low” clustering areas were found in urban areas. Thus, the process is more diffused in rural areas for the identified countries, suggesting risk factors such as physical inactivity, unhealthy dietary patterns and excessive alcohol drinking and smoking are yet to be under control or mitigated. The same processes could be driving high-high clustering in urban South Africa. For example, urban residents in South Africa take high fat and sugar content diets that are low in carbohydrates and fibres, while rural populations follow a traditional diet which is high in carbohydrates and fibre content but low in fats and sugars [23]. Over the years, a transition from rural to urban life has seen the urban majority transitioning to an urban life and diets [22,23]. Evidence has shown that a higher proportion of the urban Black population with low economic status are heavily depended on fast food [40]. Thus, dietary patterns and lifestyles may help to explain the disparities in the spatial co-clusters of CVDs and their risk factors across the districts in South Africa. There is a need for the modification of the dietary patterns of the urban population to have adequate nutrient intake to prevent increased incidence of CVDs and their risk factors.

The presence of spatial clustering in CVDs and their risk factors has also been found in different countries such as Nigeria [36], Sweden [39], France and Australia [37], and the USA [41]. However, our modelling approach has allowed us to measure the co-clustering of CVDs and risk factors. We have found that stroke and high blood cholesterol and smoking co-cluster in space, which supports the notion that stroke, tobacco smoking and blood cholesterol are positively associated [21,42,43]. 

Our findings are generally in agreement with earlier studies in South Africa that used spatial statistical methods to analyze CVDs and their risk factors. For example, Kandala et al. [29], using a Bayesian geo-additive mixed model, found a high prevalence of hypertension in north-central-western parts of the country and low prevalence in the north-eastern part of the country. Wandai et al. [30] also found significantly above average prevalence of hypertension in the districts of the north-central-western parts of the country, as revealed by this study. Darikwa et al. [31] found cardiovascular mortality to co-cluster in the south-western part of the country.

### 4.1. Strength

The strength of our study has been the novel application of the bivariate spatial autocorrelation modelling approach to measure clustering and local co-clusters of CVDs and their risk factors. Studies by Penney et al. [35], Paquet et al. [37] and Rajabi et al. [39] employed univariate spatial clustering methods. Their approaches could be limited, as CVDs and risk factors tend to co-occur at both individual and ecological levels [41]. Kandala et al. [29], noted that CVDs and their risk factors have similar aetiology such that analyzing them independently would be less efficient. In addition, estimating joint “hot spot” and a low cluster of districts for two or more CVDs will provide more evidence for an integrated intervention approach that targets all the modelled diseases instead of targeting only one CVD. Additionally, by using age–sex standardized incidence rates, our study removed the effect of age and gender, two of the major determinants of health. However, we still find pockets of high risk of CVDs and their risk factors, a finding that suggests that other risk factors could be affecting the spatial variations in CVD incidence rates. As alluded to in Mena et al. [44] and Elmadfa and Meyer [45], accessibility to health services, socio-economic factors, level of urbanity, educational level, food composition and intake of nutrients, water quality, temperature and other environmental factors could also impact on geographical variations in CVDs and risk factors. Thus, the differences in observed clustering that we have observed, even after accounting for differences in age and gender distribution across the districts, could be due to differences in these other factors, but more data would be needed to confirm this assertion.

### 4.2. Limitations

The data on high blood cholesterol, smoking, stroke and heart attack were self-reported. Newell et al. [46] noted that inaccurate self-reporting could result in the overestimation or underestimation of the disease burden. Biomarkers can be used to redress the problem but, unfortunately, these were not available. Without supporting data for validation, the results of the present study need to be treated with caution. However, self-reported values and directly measured values tend to be highly correlated, even in the presence of bias [47,48,49]. It is our conviction that, even in the presence of bias in the self-reported values, the spatial autocorrelation patterns obtained in this study would not change much when measured values were to be used. Our analyses were done at the district level, which is the level at which primary health is provided in South Africa. Aggregation of the results has the effect of introducing ecological fallacy and large geographical units of analysis may mask some information of interest [44]. Results and efficiency may be improved by having smaller units of analysis [44]. According to Paquet et al. [37], when conducting spatial epidemiology, the administrative unit to use in the analysis goes beyond just the size of the unit of analysis and will need to be studied for each given setting. Our study excludes adults older than 64 years old. This was done to focus on the spatial patterns attributable to the productive age group of 15–64 years, which overlaps with the age range in which premature mortality occurs (less than 70 years). However, it is hereby acknowledged that this limits the ability to evaluate patterns in the age groups that are at the highest risk of cardiovascular disease (65 years and greater).

## 5. Conclusions

Cardiovascular disease (CVD) is a major contributor to the health burden in South Africa. Using novel spatial clustering statistical techniques, the study identified joint spatial association and locations of similar rates of CVDs and their risk factors among adults in South Africa. Although the study findings are mostly confirmatory, they are nonetheless important in supporting the identification of priority areas for public health interventions. The finding that districts that tend to co-cluster in the urban areas have higher rates of CVDs and risk factors than districts that co-cluster in rural areas suggests that there are more contagious and spatial diffusion processes among interdependent districts in urban districts. The level of rurality of locations need to be considered when intervention initiatives are implemented. Evidence of co-clustering may point to having an integrated intervention programme targeting several CVDs and associated risk factors simultaneously, mainly in these urban districts, and might be more effective and less costly.

## Figures and Tables

**Table 1:** Summary statistics of the prevalence of cardiovascular diseases (CVDs) and related risk factors across the districts.

Sub-Group	CVD or Risk Factor	Minimum	25% Quartile	Median	Mean	75% Quartile	Max	Sample Size
All	Stroke	0.0%	0.0%	1.1%	1.2%	1.6%	9.1%	9154
	Heart attack	0.0%	1.1%	2.2%	2.4%	3.5%	9.1%	
	Smoking	0.0%	15.8%	20.8%	21.9%	25.0%	53.2%	
	HBC	0.0%	0.7%	1.5%	2.2%	3.0%	9.1%	
	Hypertension	9.5%	27.6%	32.9%	34.3%	41.2%	65.4%	
Male	Stroke	0.0%	0.0%	0.0%	1.1%	1.2%	20.0%	3817
	Heart attack	0.0%	0.0%	1.1%	1.8%	2.2%	20.0%	
	Smoking	0.0%	31.8%	37.8%	39.0%	46.1%	81.8%	
	HBC	0.0%	0.0%	0.7%	1.8%	2.9%	20.0%	
	Hypertension	0.0%	22.4%	29.6%	31.2%	40.8%	80.0%	
Female	Stroke	0.0%	0.0%	1.3%	1.3%	2.2%	5.4%	5337
	Heart attack	0.0%	1.1%	2.6%	2.8%	4.1%	10.7%	
	Smoking	0.0%	1.7%	4.8%	9.9%	12.3%	41.7%	
	HBC	0.0%	0.2%	1.6%	2.4%	2.9%	12.5%	
	Hypertension	15.4%	29.9%	35.7%	36.3%	41.6%	66.7%	
15–39	Stroke	0.0%	0.0%	0.0%	0.5%	0.8%	2.9%	5848
	Heart attack	0.0%	0.0%	0.3%	0.9%	1.4%	4.6%	
	Smoking	0.0%	14.4%	19.6%	20.1%	23.4%	46.6%	
	HBC	0.0%	0.0%	0.0%	0.6%	0.8%	5.7%	
	Hypertension	0.0%	15.3%	20.8%	21.0%	27.6%	40.9%	
40–64	Stroke	0.0%	0.0%	1.5%	2.2%	3.5%	10.0%	3306
	Heart attack	0.0%	0.6%	4.5%	4.5%	6.5%	14.1%	
	Smoking	0.0%	15.7%	21.8%	23.4%	29.6%	66.7%	
	HBC	0.0%	1.3%	3.3%	4.6%	5.7%	19.1%	
	Hypertension	25.0%	47.2%	56.8%	55.5%	63.9%	92.3%	
Male 15–39	Stroke	0.0%	0.0%	0.0%	0.1%	0.0%	2.0%	2574
	Heart attack	0.0%	0.0%	0.0%	0.7%	1.3%	5.6%	
	Smoking	0.0%	27.4%	35.7%	35.4%	44.5%	66.7%	
	HBC	0.0%	0.0%	0.0%	0.4%	0.0%	5.9%	
	Hypertension	0.0%	15.2%	21.2%	22.2%	29.7%	50.0%	
Female 15–39	Stroke	0.0%	0.0%	0.0%	0.7%	1.3%	4.4%	3274
	Heart attack	0.0%	0.0%	0.0%	1.1%	1.9%	7.3%	
	Smoking	0.0%	1.2%	3.9%	8.0%	7.9%	40.5%	
	HBC	0.0%	0.0%	0.0%	0.8%	1.1%	10.3%	
	HT	0.0%	15.2%	20.9%	19.8%	25.4%	37.7%	
Male 40–64	Stroke	0.0%	0.0%	1.2%	2.1%	2.5%	25.0%	1243
	Heart attack	0.0%	0.0%	0.0%	3.3%	5.9%	25.0%	
	Smoking	0.0%	35.0%	42.9%	45.2%	52.1%	100.0%	
	HBC	0.0%	0.0%	0.0%	3.7%	5.7%	25.0%	
	Hypertension	0.0%	36.5%	50.0%	46.9%	61.3%	100.0%	
Female 40–64	Stroke	0.0%	0.0%	1.2%	2.1%	4.7%	8.3%	2063
	Heart attack	0.0%	0.0%	4.9%	5.1%	7.6%	18.8%	
	Smoking	0.0%	0.8%	6.9%	11.5%	17.1%	46.2%	
	HBC	0.0%	0.0%	3.1%	4.9%	5.3%	27.8%	
	Hypertension	33.3%	52.1%	61.4%	60.3%	67.6%	100.0%	

**Table 2:** Global univariate and bivariate spatial autocorrelation association between the age–sex standardised incidence rates of CVDs and identified risk factors for all participants.

	Stroke	Heart Attack	Smoking	HBC	Hypertension
Stroke	0.128 *	−0.019 ^INS^	0.218 **	0.184 **	−0.075 ^INS^
Heart attack		−0.015 ^INS^	−0.099 ^INS^	−0.021 ^INS^	−0.008 ^INS^
Smoking			0.606 ***	0.366 ***	0.149 *
HBC				0.355 ***	−0.077 ^INS^
Hypertension					0.236 **

Key: HBC, high cholesterol; HT, hypertension; INS, insignificant at 5% level; *, significant at 10% level; **, significant at 5% level; ***, significant at 1% level. Shaded elements are univariate global Moran’s indexes.

## Appendix B

**Table A1:** Effects of stratification on the sample size used in the analysis.

Sub-Group	Mean	Std Error	Median	Minimum	Maximum	Range	Sample Size
All	203	20	185	23	544	544	10,336
All (15-64 years)	179	18	157	11	503	492	9154
Male (15-64 years)	75	8	58	5	252	247	3817
Female (15-64 years)	105	10	89	6	288	282	5337
Male 15-39	50	5	42	1	159	158	2574
Female 15-39	64	6	55	1	193	192	3275
Male 40-64	24	3	20	1	93	92	1243
Female 40-64	40	4	36	3	125	122	2063
